# Encapsulation of Zootechnical Additives for Poultry and Swine Feeding: A Systematic Review

**DOI:** 10.1021/acsomega.4c08080

**Published:** 2025-02-13

**Authors:** Liliana Berté Fontana, Guilherme Schwingel Henn, Carolina Horst Dos Santos, Luana Specht, Caroline Schmitz, Claucia Fernanda Volken de Souza, Daniel Neutzling Lehn

**Affiliations:** † Graduate Program in Biotechnology, Universidade do Vale do Taquari - Univates, Lajeado 95900-000, Rio Grande do Sul, Brazil; ‡ American Nutrients do Brasil Indústria e Comércio Ltda, Teutônia 95890-000, Rio Grande do Sul, Brazil

## Abstract

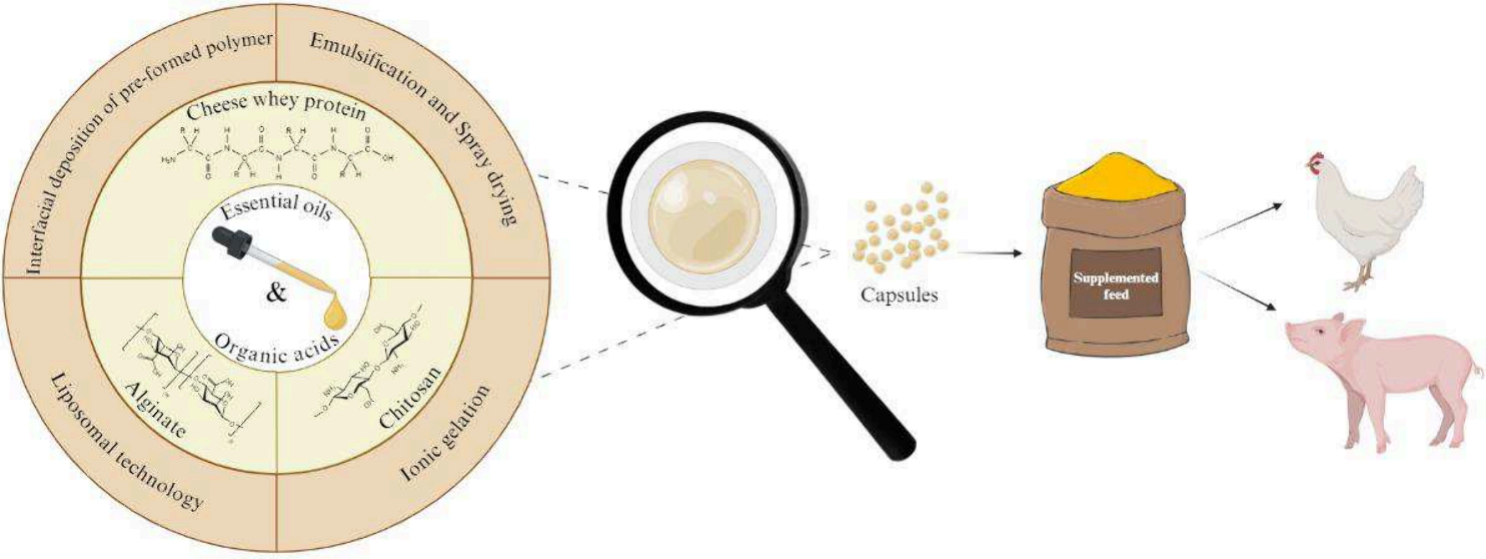

The search for alternatives to certain antibiotics in animal nutrition has propelled the study of encapsulated essential oils and organic acids considering their potential to generate beneficial effects in animal organisms. The objective of this study was to compile and discuss scientific findings published between 2013 and July 2024 from two databases related to the usage of encapsulated essential oils and organic acids in the supplementation of poultry and swine feeds. A systematic review was conducted following the Preferred Reporting Items for Systematic Reviews and Meta-analyses (PRISMA) methodology, covering the PubMed and Web of Science databases, which initially yielded 115 selected articles. After applying the inclusion and exclusion criteria, 21 relevant articles were selected for comprehensive analysis. The studies demonstrate that the encapsulation of essential oils and organic acids is an alternative to reduce the utilization of conventional antibiotics, as encapsulation has the potential to maintain the properties of these compounds while ensuring greater stability and controlled release within the animal organism. The selection of appropriate encapsulation technologies, encapsulating agents, and zootechnical additives is crucial to maximizing the effectiveness of these compounds in animal nutrition. Despite the identification of gaps in the analyzed studies regarding specific details of the techniques used and regulatory considerations, encapsulated essential oils and organic acids show potential to reduce the need for antibiotics in animal production along with other added benefits. This Review provides a comprehensive overview of the subject, aiming to guide and contribute to future research efforts.

## Introduction

In livestock systems, the routine use of antibiotics to prevent disease and enhance feed efficiency has raised growing public concern due to the emergence of antibiotic-resistant bacteria, which can pose risks to human health.^1−3^ In this context, zootechnical additives—substances used to positively influence the performance of livestock—have emerged as promising alternatives. Among these additives, phytogenics, such as essential oils and organic acids, have gained prominence. These compounds possess antimicrobial, anti-inflammatory, antiviral, antioxidant, and immunomodulatory properties, and they help regulate the gastrointestinal tract, improving nutrient digestibility and absorption.^1−7^ However, despite their benefits, these compounds still face significant limitations in their effective large-scale application.

One of the main challenges is the chemical instability of essential oils, which are highly volatile and susceptible to degradation when exposed to heat, light, and oxygen.^7−9^ Additionally, many of these compounds have strong flavors and odors, which may hinder feed and water intake, directly impacting animal performance.^8^ Lastly, the rapid and uncontrolled release of active compounds in the digestive tract diminishes their efficacy, limiting their therapeutic effects.^9^

In this context, the encapsulation of bioactive compounds has emerged as a promising strategy to overcome these limitations through the controlled release of these compounds and the preservation of their properties. Encapsulation is a process involving the coating of small particles (e.g., the active substances) with a wall material, producing capsules that function as physical barriers to prevent or stall the occurrence of undesirable physical and chemical reactions.^7,10^ This protective technology proves to be highly versatile and adaptable to various needs and can be classified as physical, chemical, or physicochemical depending on the principle behind capsule formation.^11^ Encapsulation allows the use of a variety of raw materials and encapsulating agents of animal or plant origin in combination with the precise application of specific methods, playing a fundamental role in various manufacturing processes.

The purpose of this study is to compile and discuss scientific results published between 2013 and July 2024 related to the use of encapsulated essential oils and organic acids in the dietary supplementation of poultry and swine. Part of the aim of this work is to identify which essential oils and organic acids are promising for improving zootechnical performance as well as to analyze the techniques and encapsulating agents most frequently used, taking into consideration their potential for industrial-scale applications. To the best of our knowledge, no systematic review of this topic has been conducted to date. The choice of poultry and swine as subjects was due to their shorter production cycles compared to species such as cattle as well as being raw materials for some of the most produced and consumed meats worldwide. The central hypothesis is that natural additives can be effective alternatives to antibiotics traditionally used in the livestock industry.

## Methodology

**Figure 1:**
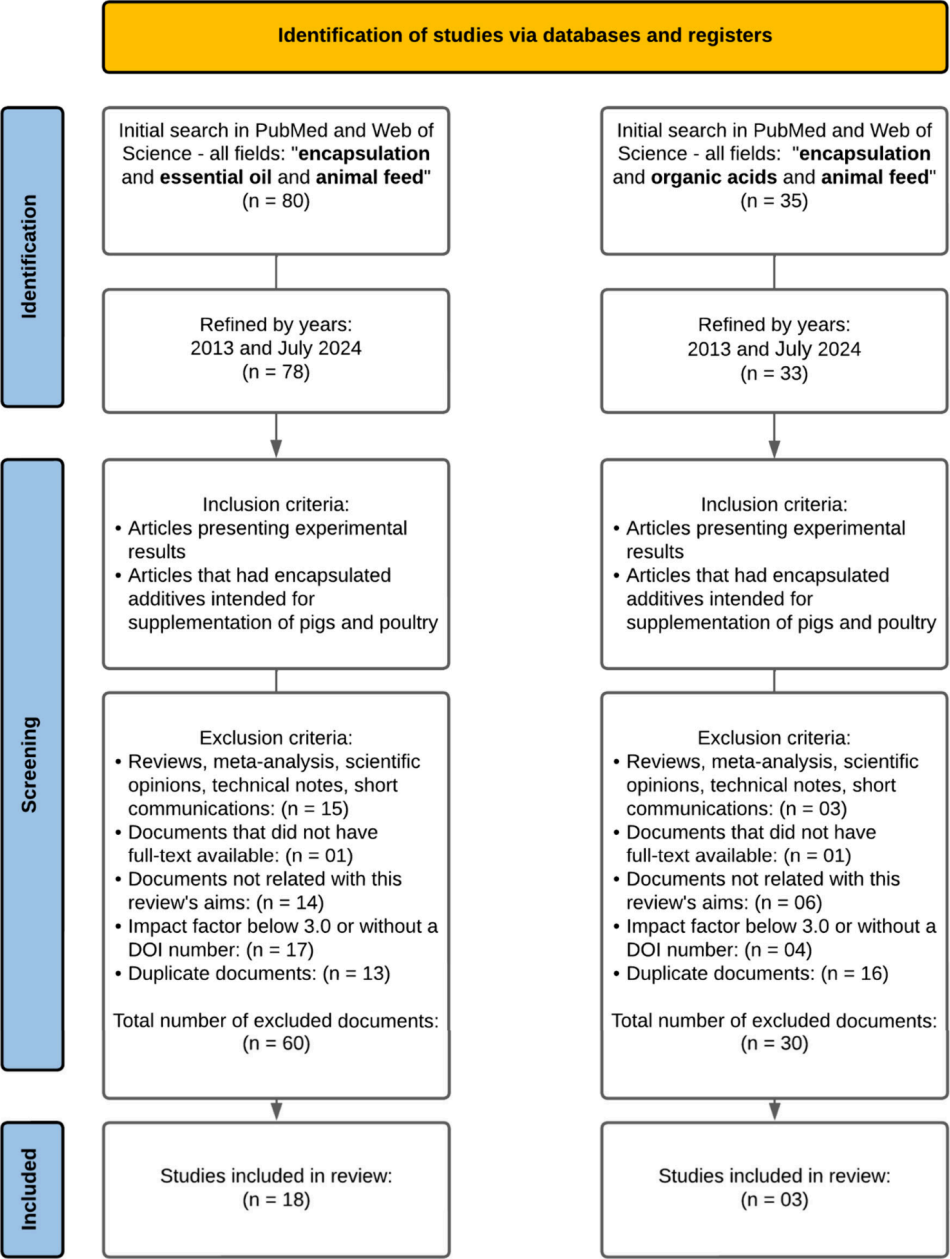
Outline of the systematic review process.

The Preferred Reporting Items for Systematic Reviews and Meta-analyses (PRISMA) methodology^12^ was employed to structure this systematic review. This approach aims to help systematic reviewers transparently report why the review was conducted, the methods used, and the findings obtained. A literature search was conducted using two databases, PubMed and Web of Science. Figure 1 provides a detailed description of the selection process for the articles included in this review. The keywords used in the searches were divided into two groups based on the scope of this review: (a) “encapsulation”, “essential oil”, and “animal feed” and (b) “encapsulation”, “organic acids”, and “animal feed”.

After the literature search, the documents underwent meticulous analysis, beginning with refinement according to the year of publication, wherein only articles published between 2013 and July 2024 were selected. Certain types of articles (specifically reviews, meta-analyses, scientific opinions, technical notes, and short communications) were excluded, as they lacked comprehensive information or methods and were therefore deemed outside the scope of this review. Further exclusions included documents without full-text access and duplicate entries. To ensure only high-quality articles were assessed, those lacking a Digital Object Identifier (DOI) or published in journals with an Impact Factor below 3.0 were also excluded from the search.

In the inclusion stage, we selected articles presenting experimental results, and the resulting list from the bibliographic database search was manually reviewed based on the scope of this work. This was done by assessing whether the article title and abstract description were related to the study’s objectives, specifically encapsulated additives intended for poultry and swine feed supplementation.

The selected articles were read in their entirety, and relevant information was extracted through the following inquiries. (I) What technique was used? (II) What encapsulating agents were employed? (III) What additives were used? (IV) Were *in vitro* experiments and/or *in vivo* experiments conducted with poultry and/or swine? (V) What were the main findings? The data were organized, tabulated, and discussed, ensuring greater reliability of the review results.

## Results

The initial query using the selected keywords in the PubMed and Web of Science databases yielded a total of 115 documents. After applying the aforementioned inclusion and exclusion criteria, 21 articles were selected for detailed analysis, as illustrated in Figure 1. This rigorous selection aims to ensure the quality and relevance of the studies included in this review.

**Figure 2:**
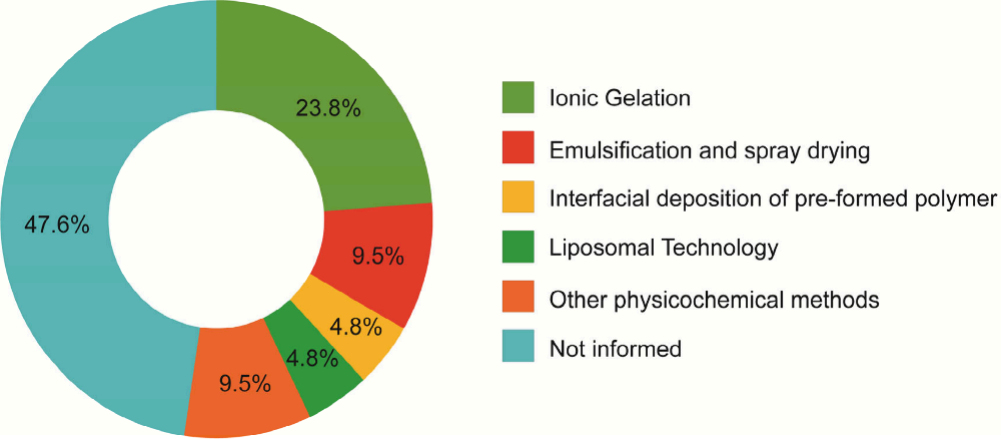
Encapsulation techniques and the percentage of studies conducted employing each method.

Among the selected articles, a significant number (*n* = 10) did not provide enough information on the encapsulation techniques employed. However, among those that did provide such information, the Ionic Gelation technique was the most prevalent, as depicted in Figure 2.

Within the examined articles, 38% did not mention the encapsulating agents employed. However, among those that did provide this information, the most frequently utilized capsule coating materials were alginate (25%), chitosan (10%), and cheese whey protein (10%). The compounds commonly used in the analyzed articles include thymol, carvacrol, and citral, which are phytoconstituents present in several essential oils, and organic acids including sorbic, fumaric, benzoic, butyric, and hexanoic acids.

Among the articles analyzed in this systematic review, there was a predominance of studies which tested the produced capsules *in vivo*, totaling 14 articles,^5,8,13−24^ compared to 2 articles which explored only *in vitro* assays,^7,25^ 3 articles reporting a combination of both *in vitro* and *in vivo* experiments,^26−28^ and 2 articles did not conduct any tests.^29,30^ Additionally, studies prioritized the use of poultry as an animal model, with 15 occurrences among the analyzed articles.^5,7,8,13,15,17−19,21−24,26−28^ Only one of the reviewed works did not employ any form of simulation. Table 1 presents essential information obtained from the 21 selected articles, including encapsulation technologies, the methods used for encapsulation, the encapsulation agents, the zootechnical additives incorporated, simulation of the gastrointestinal tract of swine or poultry, and finally, the main findings drawn from each article.

**Table 1:** Selected Articles on the Review Topic after Database Search Containing Key Information of Interest

Encapsulation Technology	Encapsulation Method	Encapsulating Agents	Zootechnical additive	(Simulated) Gastrointestinal Tract^a^	Main Findings	Reference
Emulsification and spray drying	Physicochemical	Soy protein isolate, soy polysaccharides, maltodextrin, and soy meal	Cinnamon essential oil	Poultry - *In vivo* (*n* = 126, 180) assay	Dietary supplementation with encapsulated cinnamon essential oil reduced mortality and condemnation rates in poultry	(24)
					Improvement in intestinal health with a reduction in necrotic enteritis and an increase in the villus height/crypt depth ratio in the jejunum	
					Assistance in overcoming heat stress	
					Efficacy as an alternative to antibiotics in commercial poultry production	
Emulsification and spray drying	Physicochemical	Soy protein isolates and soluble polysaccharides; Soy-derived polymer	Citral - compound supplied by Sigma-Aldrich	Poultry - *In vivo* (*n* = 216) and *in vitro* assays	The employed encapsulation technique has application in the large-scale production of encapsulated essential oils or other lipophilic alternatives to antibiotics in animal feed	(27)
					Encapsulated citral inhibited the growth of *Clostridium perfringens* by more than 4× compared to unencapsulated citral and could be used to control Necrotic Enteritis in poultry	
Extrusion and ionic gelation	Chemical	Alginate and cheese whey proteins.	Carvacrol - compound supplied by Sigma-Aldrich	Poultry - *In vivo* (*n* = not informed) and *in vitro* assays	Without adequate protection, carvacrol does not reach the lower gastrointestinal tract	(26)
					Encapsulation prevents the reduction of carvacrol in the upper gastrointestinal tract and increases its concentration in the lower gastrointestinal tract	
					*In vivo* release of carvacrol appeared faster than release in the *in vitro* simulation	
					Encapsulation efficiency percentage ≥98%	
Ionic gelation	Chemical	Sodium alginate and calcium chloride	Thyme essential oil - obtained via the process of hydro distillation with steam flow at countercurrent into a rotating cone column	None	Essential oil content is a determining factor in the size and shape of the microspheres	(30)
					Higher degrees of essential oil dispersion improve the encapsulation efficiency and loading capacity of the microspheres	
					The obtained microspheres showed a significant antimicrobial effect, especially on Gram-positive bacteria	
					Encapsulation efficiency: 85%.	
Ionic gelation (Melt-granulation process)	Chemical	Starch and alginate	Thymol, lauric acid, palmitic acid, stearic acid - compounds supplied by Sigma-Aldrich	Swine - *In vitro* assay	Among the three acids tested, lauric acid was selected for further studies because its mixture with thymol remained a homogeneous liquid at room temperature for 6 h	(25)
					The formulation and method established in this study for the encapsulation of thymol and lauric acid are relatively simple and can be potentially used as a method for the effective delivery of essential oils and medium-chain fatty acids to the intestinal tract of pigs	
					Thymol and lauric acid had good stabilities (>90%) in both types of microparticles with and without alginate	
					Microparticles produced with alginate had better compound release parameters in the intestine, while particles without alginate showed rapid release of bioactive compounds after incubation in simulated salivary fluid	
Melt-solidification technique	Chemical	Microcrystalline cellulose, magnesium aluminum, wheat bran	*trans*-cinnamaldehyde and eugenol - obtained from Sigma-Aldrich	None	Cinnamaldehyde encapsulated with lauric acid showed better inhibitory activity over palmitic acid-based granules and free cinnamaldehyde	(29)
					The encapsulation efficiency was 93%	
					The formulations and method developed in this work for encapsulation of oils in solid granules are relatively simple and economical and can potentially be used to encapsulate a variety of pure essential oils and their mixtures or other lipophilic liquid actives	
Cinnamaldehyde: melt-solidification technique; Citral: oil-in-water emulsions	Chemical	Cinnamaldehyde: Adsorbent powder and fatty acid; Citral: Soy protein isolate and soluble soy polysaccharide	Cinnamaldehyde and citral were encapsulated separately - compounds supplied by Sigma-Aldrich	Poultry - *In vivo* (*n* = 3,200) assay	Encapsulated cinnamaldehyde and encapsulated citral (alone or in combination) had similar results to the antibiotic bacitracin, and resulted in significantly improved body weight, altered composition of cecal microbiota, improved intestinal health, and increased growth performance in poultry	(8)
Ionic gelation	Chemical	Chitosan derived from crab shells and sodium triphosphate pentabasic (TPP)	Garlic essential oil - supplied by Exir Pharmaceutical Company	Poultry - *In vivo* (*n* = 900) and *in vitro* assays	Nanoencapsulated essential oils showed greater benefits than those in free form	(28)
					Nanoencapsulated garlic essential oil inhibited the growth of Escherichia coli by more than 8× in comparison to the nonencapsulated form	
					Garlic essential oil possesses antibacterial and antioxidant properties and aids the intestinal health and performance of poultry, leading to weight gain and increased population of *Lactobacillus*	
					Possible alternative to antibiotics as growth promoters in poultry production	
					The encapsulating agent chitosan is effective and inexpensive	
Ionic gelation (Sharp-hole condensation method)	Chemical	Sodium alginate and chitosan	Cinnamon, thyme, and peppermint essential oils - supplied by Shanghai McLean Biochemical Technology Co., Ltd.	Poultry - *In vitro* assay	Essential oils are potential substitutes for antibiotics	(7)
					Anti-inflammatory, antioxidant, bacteriostatic, and antiviral effects	
					The amount of compound released from the microparticles was greater in the simulated intestinal fluid than in the simulated gastric fluid, which is in line with the organism’s digestive and absorptive characteristics	
					Encapsulation efficiency: 80.33 ± 2.35%	
					Average dry particle size: around 0.8 mm.	
Not specified	Not specified	Not specified	Carvacrol, thymol, and limonene - capsules were supplied by BIOMIN Holding GmbH	Poultry - *In vivo* (*n* = 600) assay	The benefits of supplementing poultry diets with a mixture of encapsulated essential oils were superior to those of the phytogenic additives in powder form	(13)
					Addition of encapsulated essential oils improved performance as well as the apparent ileal digestibility of nutrients in poultry, possibly due to improved secretion of digestive enzymes	
Not specified	Not specified	Not specified	Phenolic compounds, target release butyrate, short-chain fatty acids, formic acid, acetic acid, and propionic acid - supplier not given	Swine - *In vivo* (*n* = 240) assay	Dietary supplementation with organic acids did not improve growth performance, while intestinal health was improved via increased levels of short-chain fatty acid and lower levels of *E. coli*	(14)
					Organic acid mixtures promote similar growth to antibiotics	
Not specified	Not specified	Not specified	Sorbic acid, fumaric acid, and thymol - capsules supplied by Jefo Nutrition Inc.	Poultry - *In vivo* (*n* = 144^5^ and *n* = 504^15^) assay	Encapsulated essential oils and organic acids act as growth promoters, improving feed conversion and overall animal performance	(5, 15)
					They support intestinal health by increasing *Lactobacillus* spp. cell counts, maintaining intestinal morphology, and enhancing digestive, absorptive, and barrier functions	
					They reduce harmful bacteria such as *Escherichia coli* while increasing short-chain fatty acid concentrations and the activity of digestive enzymes, lowering disease risks	
					These compounds exhibit antimicrobial activity comparable to the antibiotic enramycin, without adverse effects on beneficial bacteria	
Not specified	Not specified	Not specified	Thymol and carvacrol - capsules supplied by EUGENE BIO Co.	Poultry - *In vivo* (*n* = 600) assay	Essential oils can be used as an alternative to anticoccidial agents to mitigate coccidiosis-induced growth depression in poultry	(19)
					The use of the antibiotic salinomycin was compared with encapsulated essential oil in the diet of poultry	
					Essential oils exhibit antioxidant effects and reduce body temperature and volatile fatty acid concentrations in poultry	
Not specified	Not specified	Hydrogenated vegetable oil triglyceride matrix	Fumaric, citric, malic, and sorbic acids and thymol, vanillin, and eugenol–capsules were supplied by Jefo Nutrition Inc.	Swine - *In vivo* (*n* = 30) assay	Essential oils and organic acids microencapsulated in combination with antibiotics improved performance, growth, ability to mitigate inflammation, and exhibit an immunomodulatory effect	(16)
					Increased feed efficiency, protection of the intestinal barrier by increasing tight junction protein expression and increasing the abundance of *Lactobacillus* and *Bacilli*	
					Increased feed efficiency, protection of the intestinal barrier, enhanced tight junction protein expression and *Lactobacillus* and *Bacilli* abundance.	
					Free acidifiers combined with antibiotics showed a weaker effect on animal growth and intestinal health compared to the microencapsulated organic acids and essential oils additive	
Not specified	Not specified	Calcium palmitate	Tributyrin with oregano essential oil or methyl salicylate - capsules supplied by Lucta (Guangzhou) Flavours Co., Ltd.	Swine - *In vivo* (*n* = 108) assay	The encapsulated mixture of methyl salicylate and tributyrin showed more significant results compared to the encapsulated mixture of tributyrin and oregano essential oil	(20)
					The encapsulated mixture of methyl salicylate and tributyrin improved growth performance of piglets, antioxidant capacity, and intestinal villus morphology and modulated the microbiota and its metabolites, making it an efficient feed additive for piglets	
Not specified	Not specified	Calcium alginate and cheese whey proteins, Not specified, and Palm oil, respectively	Thyme, carvacrol, hexanoic acid, benzoic acid, butyric *acid -* capsules were supplied by Menon Animal Nutrition Technology.	Poultry - *In vivo* (*n* = 240,^17^*n* = 432^21^ and *n* = 228^22^) assay	Supplementation with essential oils and organic acids reduces intestinal damage such as necrotic enteritis, decreases systemic and mucosal inflammation, improves feed conversion, modulates the microbiota, and increases intestinal goblet cells, strengthening the intestinal barrier function	(17, 21, 22)
					Promotes the growth of beneficial microorganisms (*Lactobacillus*, *Enterococcus*, *Faecalibacterium*, *Bacteroides*) and reduces pathogens (*Erysipelotrichaceae*, *Ruminococcus*, *Escherichia coli*)	
					This approach presents a promising alternative to antibiotics	
Interfacial Deposition	Chemical	Not specified	Glycerol monolaurate (composed of lauric acid and glycerol) - capsules supplied by Seebio Biotech, Inc.	Poultry - *In vivo* (*n* = 84) assay	Nanoencapsulated organic acids at the highest dosage improved the health of poultry, mainly through the stimulation of glutathione S-transferase (GST) activity, enzyme responsible for liver detoxification	(18)
Liposomal encapsulation	Physicochemical	Liposome	Oregano, cinnamon, and clove essential oil - capsules supplied by Leda Medical	Poultry - *In vivo* (*n* = 200) assay	Essential oils encapsulated exhibited antioxidant and antibacterial properties and improved tight junction proteins, intestinal barrier functions, and digestive enzymes at serum and molecular levels	(23)
					Improved body weight gain and feed conversion rate in poultry fed with higher levels of essential oils	
					Abundance of beneficial bacteria, as well as an increase in bacterial metabolites (valeric acid, butyric acid, propionic acid, acetic acid, and total short-chain fatty acids), while that of pathogens was reduced	

a *n* = number of animals used in *in vivo* experiments.

## Discussion

The analysis of the 21 selected articles indicates that the use of encapsulated essential oils and organic acids in feed supplementation for meat-producing animals is justified by various factors and benefits. These additives contribute to more natural food production. They serve as prophylactic agents, enhancing immunity, mitigating performance drops, and potentially reducing or even replacing traditional antibiotics in livestock production.^3,22,31−33^

**Figure 3:**
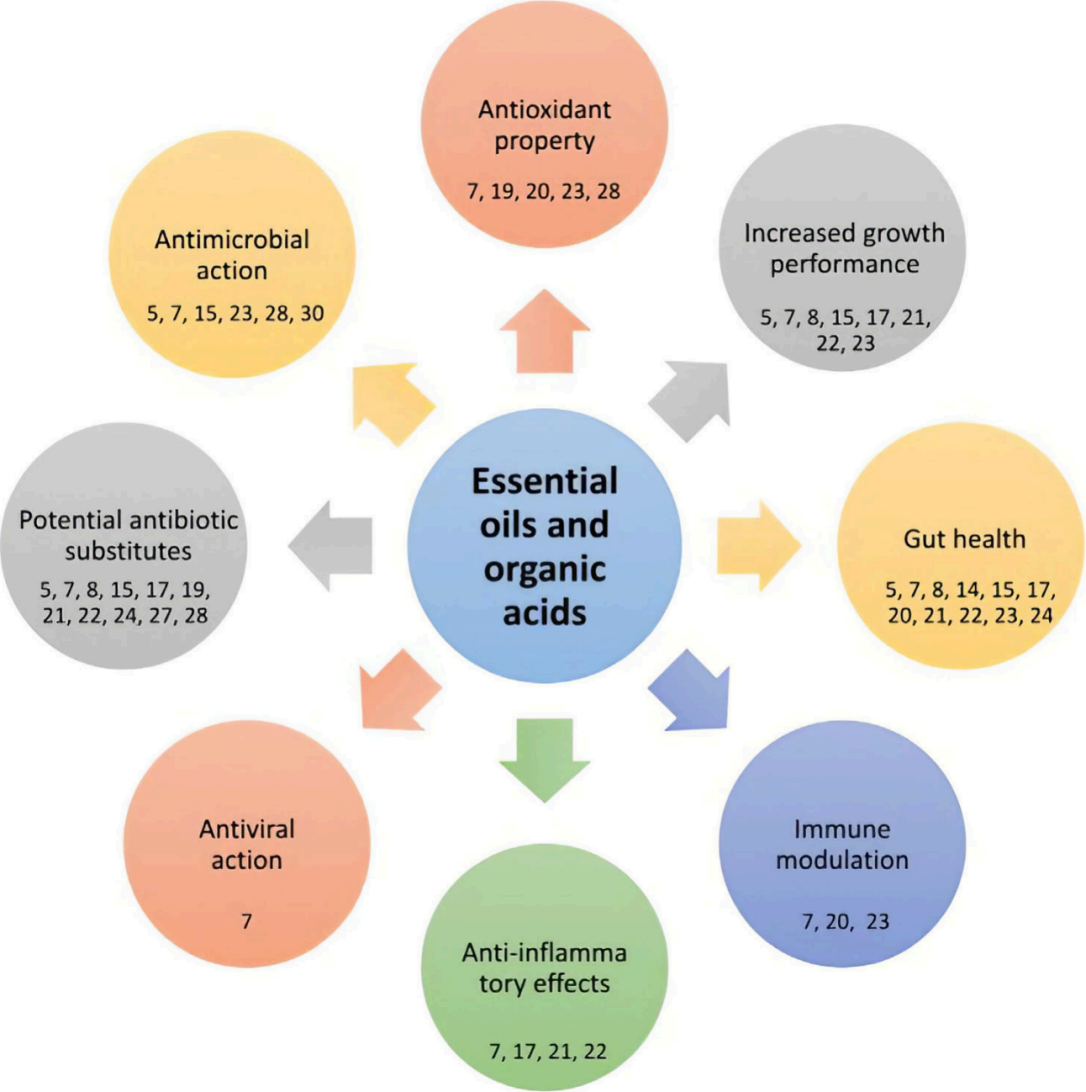
Potential effects of essential oils and organic acids in the organisms of swine and poultry.

Figure 3 presents the potential effects of essential oils and organic acids, as reported in the studies included in this systematic review. These compounds exhibit antioxidant effects, promote growth, and enhance intestinal health. They also show immunomodulatory effects, anti-inflammatory properties, antimicrobial and antiviral activities, and the potential to serve as substitutes for traditional antibiotics.^7,16,19,20,22,23,28^ Furthermore, they can reduce body temperature and concentrations of volatile fatty acids, promoting higher levels of total short-chain fatty acids and the activity of digestive enzymes.^19,23^ These properties help decrease the risk of diseases such as necrotic enteritis and coccidiosis in poultry.^17,19,24,34^

An experimental study that coinfected birds with *Eimeria* and *Clostridium perfringens* to evaluate the potential of essential oils and organic acids in controlling necrotic enteritis resulted in effective control over this infection.^17^ Furthermore, evidence suggests that the use of vaccines against coccidiosis can be complemented by the inclusion of essential oils in the diets of poultry. One study indicated that adding essential oils to the diets of broiler chickens vaccinated against coccidiosis mitigated the vaccine-induced depression in weight gain and feed intake, without affecting the feed conversion ratio.^19^ This indicates that essential oils can be an effective strategy for overcoming the negative effects associated with immunological challenges, increasing feed intake and consequently promoting growth and overall health in birds. Thus, incorporating these compounds into their diet as feed additives is recommended, showing promising economic efficiency.^23^ This approach can not only enhance the performance of broiler chickens but also contribute to more sustainable production practices that are less reliant on antibiotics.

A wide range of essential oils and organic acids was found in the analyzed articles, highlighting the variety of options available for animal diet supplementation. These compounds may be used individually or in combinations; when two or more active agents are combined, they may interact with the animal organism in different ways, resulting in four possible effects: synergy, partial synergy, indifference, or antagonism.^35^ Some studies address the synergistic effects between organic acids and phytogenic compounds leading to effective reduction in pathogenic bacteria cell count.^5,17,22,36−38^ This approach has received significant attention due to the potential synergistic benefits in growth performance and health in swine and poultry when compared to the effects of individual compounds.^17^

Thyme essential oil and its major constituents, thymol and carvacrol, along with citral, a compound found in lemongrass, lemon, and other citrus essential oils, have been the most studied bioactives in poultry and have shown a high potential for antimicrobial,^27^ antifungal,^39^ and antiparasite activity.^8^ These bioactives are terpenes considered natural volatile compounds and are already marketed in commercial encapsulated products.^16,17,22^

Carvacrol (5-isopropyl-2-methylphenol) is a monoterpenoid phenolic compound, and its antimicrobial activity is directly related to its concentration, where it is reported that at a concentration of less than 1000 ppm it has no antimicrobial activity on *Salmonella* and *Escherichia coli*.^26^ The effect of carvacrol and thymol tends to alter the ileal pH toward an acidic state, which supports the reduction of *E. coli* in broilers.^40^*In vitro* and *in vivo* studies demonstrate that when ingested, carvacrol is rapidly absorbed, resulting in low concentrations in the regions where these microorganisms are found, such as the duodenum and jejunum of swine.^26^ The encapsulation of the carvacrol molecule with materials such as whey proteins and alginate resulted in a significant increase in the molecule’s resistance, which was observed to last for up to 3 h following ingestion by poultry.^26^

Thymol (2-isopropyl-5-methylphenol) is a bioactive member of the phenolic class. In addition to the antimicrobial activity described, it has antioxidant capacity, capable of neutralizing free radicals. The antioxidant action is of particular importance for the immune system of poultry, as the excessive formation of reactive oxygen species has the potential to result in tissue damage and the disruption of physiological functions within cells, ultimately reducing the survival of the animal.^41^ Liposomal encapsulation of oregano, cinnamon, and clove oils revealed a significant improvement in body weight gain and feed conversion ratio in poultry fed 400 mg/kg of the basal diet.^23^

Supplementation with combinations of compounds, including carvacrol, thymol, and limonene, has been shown to improve poultry performance and ileal digestibility.^13^ These compounds primarily function by strengthening the intestinal barrier and modulating the microbiota, which in turn supports better regulation of immune responses in the gut.^17,21−23^ These effects may be linked to the compounds’ ability to promote the growth of beneficial bacteria, such as *Lactobacillus* spp., while inhibiting intestinal pathogens like *Salmonella* spp. and *Escherichia coli*. Additionally, maintaining the integrity of the intestinal barrier enhances nutrient absorption, positively impacting the overall health and productivity of poultry. In one study, cinnamaldehyde encapsulated with lauric acid demonstrated superior inhibitory activity compared to palmitic-acid-based granules and free cinnamaldehyde. Furthermore, a reported encapsulation efficiency of 93% encourages its potential use for controlling enteric pathogens in animals.^29^

Citral (3,7-dimethyl-2,6-octadienal), a monoterpene found in citrus essential oils, is a isomeric mixture of two aldehydes, geranial and neral.^42^ Research indicates that these compounds are destabilized in the gastrointestinal tract of poultry, which limits their antimicrobial effectiveness.^27^ To overcome this limitation, the use of protein wall materials or polysaccharides derived from the Maillard reaction has proven effective in providing adequate protection to the molecule, preventing its premature degradation, and enhancing its stability throughout the digestive system.^27^ Encapsulated citral showed significantly higher inhibition of *Clostridium perfringens*, with antimicrobial effects more than 4× stronger than nonencapsulated citral, making it an effective approach for controlling necrotic enteritis in poultry.^27^ Furthermore, encapsulated citral administration notably reduced poultry mortality rates compared to the control group, with pathogen control results comparable to those seen in vaccinated birds.^8,27^ These findings reinforce the potential of encapsulation to boost the efficacy of natural bioactive compounds as an alternative to conventional antibiotics in poultry production.

In swine, essential oils and organic acids were tested in a mix of compounds, which makes it difficult to understand the isolated effects of each compound. In addition, of the four studies that tested these animals, three used commercial products without describing the encapsulation methods, i.e., considering the animals that did not consume the encapsulated products as the control group. In general, those encapsulated with a mix of compounds provided the pigs with corresponding changes in nutrient metabolism and in the abundance of the genus of the ileum microbial community^14,20^ and increased relative abundance of *Lactobacillus* in the ileum, cecum, and colon and attenuation of inflammation.^16^

It is evident that encapsulated essential oils and organic acids provide superior benefits in animal supplementation compared to their nonencapsulated, free-form counterparts.^13,16,28^ This is because the encapsulation of volatile compounds protects them in the gastrointestinal environment of animals, providing a physical barrier against harsh conditions.^43^ Additionally, encapsulation allows for the controlled release of bioactive compounds, resulting in greater efficacy in targeting specific organs, contributing to improved health, animal performance, and food safety, allowing for their use in lieu of antibiotics or decreasing necessary dosages.^5,7,14−16^ In one study, nanoencapsulated essential oils demonstrated effects even more pronounced than those in the free form. For instance, nanoencapsulated garlic essential oil inhibited over 8-fold the growth of *E. coli* compared to its nonencapsulated counterpart.^28^

Encapsulation techniques can be classified into three distinct groups considering the principle behind the formation of capsules: (I) physical methods, which encompass processes such as spray drying, supercritical fluid precipitation, lyophilization, and solvent evaporation; (II) chemical methods, such as molecular inclusion complexation and interfacial polymerization; (III) physicochemical methods, which include techniques such as coacervation, liposomal encapsulation, and ionic gelation.^11^ However, despite the diversity of proposed techniques, a singular method has not been established as the standard for the encapsulation of active compounds.^44^ Thus, the choice of encapsulation technique depends on the desired particle or capsule size, intended application, release mechanism, and the physicochemical properties of the active agents and encapsulating materials.^45^

Among the encapsulation techniques evaluated in this review, ionic gelation is the most prominent,^7,8,25,26,28,30^ suggesting its efficacy and adaptability in encapsulating bioactive compounds for animal feed supplementation. Ionic gelation is a physicochemical extrusion method that involves the formation of gelatinous structures through the emulsification of different phase combinations, such as water/oil, oil/water, and oil/oil.^46^ This process occurs by mixing the bioactive substance with the encapsulating material and passing the resulting emulsion through an extrusion system. The resulting droplets come into contact with a solidifying solution, producing capsules that encase the bioactive substance.^47,48^

However, to enable the replicability of the ionic gelation technique on an industrial scale, the adaptation of customized equipment according to specific demands is essential considering the materials required. Alternatively, the combined technology of emulsification followed by spray drying facilitates the adaptation for large-scale production of encapsulated essential oils or other lipophilic alternatives to antibiotics in animal feed.^24,27^ It is worth noting that the lack of detailed information about additives and techniques in studies can limit a comprehensive understanding of the encapsulation processes.

The assessment of the encapsulation efficiency represents a crucial element in evaluating the quality of microcapsules. This involves determining the amount of bioactive compound effectively contained in the produced capsules.^49^ Encapsulation efficiency is influenced by various factors, such as the wall-to-core ratio, temperature, and encapsulation time.^50^ An increase in the essential oil content not only substantially reduces encapsulation efficiency but also influences the dimensions and configurations of the microcapsules.^30^ Furthermore, the analysis of capsule stability over the storage period emerges as a crucial factor to be taken into account for feed additives.^25,28^ This stability varies according to the physicochemical profile, structural parameters, and techniques employed in the process. Therefore, maintaining a high efficiency and stability is essential to ensure the effective delivery of bioactive compounds during animal feed supplementation.

Various types of polymers (encapsulating agents) have been used in the encapsulation of bioactive compounds in animal feed. The choice of an appropriate polymer plays an essential role in the efficiency and stability of the capsules, taking into consideration the properties of the encapsulated compound. The variety of available coating options reflects the constant search for effective and economical alternatives for encapsulation. Among different materials, notable ones include cheese whey and cheese whey permeate,^17,26^ alginate,^25^ starch,^25,51^ and chitosan.^28^ These materials are widely accepted due to their natural, biodegradable, and edible characteristics, making them suitable for applications in animal feed.

The use of alginate as a coating material allows for the controlled release of encapsulated compounds in the intestinal tract.^26^ This phenomenon is attributed to the nature of alginate particles, which readily decompose in alkaline environments, resulting in the maintenance of capsule integrity in the acidic gastric fluid and the release of compounds in alkaline intestinal fluid.^7^ Conversely, microcapsules that do not contain alginate in their formulation exhibit immediate release in the first part of the digestive system (the mouth), highlighting the importance of its inclusion in formulations aimed at targeted release in the intestinal tract.^25^

The technique of extrusion and ionic gelation, when combined with alginate and whey protein to encapsulate the bioactive compound carvacrol, demonstrated promising results, achieving encapsulation efficiencies of 98%.^26^ Similar efficiency results are found utilizing the same physicochemical method and the alginate polymer, but in combination with other polymers and bioactive substances. In one study, microparticles produced with or without alginate exhibited high stability (>90%).^25^ However, only microparticles produced with alginate demonstrated optimal compound release parameters in the intestine, while particles lacking alginate showed rapid release of their contents immediately upon incubation in artificial saliva solution.^25^ Other formulations achieved encapsulation efficiencies of 85%^30^ and 80%,^7^ depending on specific polymer and bioactive compound combinations. These findings underscore the robustness of alginate-based encapsulation, which allows for varied formulations tailored to the intended application.

Regarding the testing of encapsulated essential oils and organic acids in *in vitro* and *in vivo* models of poultry and pigs, determining the degree of success of the protective and release mechanisms of bioactive compounds in target organs is important to ensure their effectiveness in the animal organism. The results of studies incorporating these stages suggest growing interest in evaluating the performance of encapsulated compounds in the real physiological environment, demonstrating the practical applicability of these controlled release systems. According to the systematic review, *in vivo* tests with poultry are more frequent.^5,8,13,15,17−19,21−23,27,28^ This may be due to the smaller size of these animals, facilitating translocation and handling, and their rapid life cycle (usually 40–50 days), which allow for the quick verification of the effectiveness or lack thereof of the product.

Some studies explored the evaluation of organic acids^8,14^ and phytogenic compounds.^7^ Additionally, there were investigations into the combination of both classes of substances^21,22^ and their association with antibiotics.^16^ In this scenario, essential oils and organic acids stand out as promising options to replace antibiotics, as evidenced by recent studies.^7,22^ Their effects include the improvement of feed conversion rates,^5^ promotion of intestinal health, enhanced animal performance, and contribution toward the improvement of cecal microbiota composition by eliminating pathogens and favoring beneficial bacteria.^8,21^ These substances have shown results comparable to the use of antibiotics such as bacitracin,^8^ salinomycin,^19^ and enramycin.^5^ These findings indicate the efficacy and safety of these compounds in replacing antibiotics in the livestock and meat industry.

This review identified gaps in existing knowledge on the subject, including a lack of detailed and reproducible information, the absence of data on techniques, materials, and concentrations used, and the absence of a standardized methodology for potential studies on encapsulation of compounds. This lack of detail may be due to the need for confidentiality in some of the analyzed studies. Furthermore, despite most essential oils being Generally Regarded as Safe (GRAS) for consumption by the Food and Drug Administration (FDA),^52^ at present, no United States regulatory agency certifies or approves essential oils in terms of their quality or purity.^53^ Furthermore, regulations for these food products vary depending on the specific country in which they are commercialized,^53^ underscoring the need for standardized norms on the use of additives and quality control in the manufacturing process to ensure safety and efficacy. It is essential to rigorously evaluate them and to respect recommended doses when informed, in order to avoid potential adverse effects such as acute toxicity and the development of bacterial resistance.^54^ According to the list of ingredients and vehicles that may be used in animal feed and authorized by the Brazilian Ministry of Agriculture, Livestock, and Supply, essential oils are classified as flavorings, while organic acids are classified as preservatives and acidity regulators, with no recommendation for ingestion.^55^

Considering the above, encapsulation emerges as a viable solution to preserve unstable, volatile compounds, including essential oils and organic acids, when these are exposed to the gastrointestinal tract of poultry and pigs. Encapsulated substances can exhibit controlled release in targeted locations, presenting an opportunity for advancements in animal supplementation. Among the gaps to be addressed by future research on the topic, there is a need for more *in vivo* studies as well as the exploration of a greater variety of encapsulating materials. It is also important to compare the performance of these new compounds with existing and commonly used antibiotics, in addition to evaluating the quality of the meat obtained from animals whose feed was supplemented with encapsulated additives.

## Conclusions

This systematic review highlights that animal feed supplementation with zootechnical additives, particularly encapsulated essential oils and organic acids, is a promising strategy to enhance the productivity of poultry and swine. The analyzed substances show potential as prophylactic agents capable of boosting immunity, promoting gut health, and serving as alternatives to reduce the dependence on traditional antibiotics. Antioxidant, anti-inflammatory, and antimicrobial effects were also reported across the reviewed studies along with additional benefits such as improved feed conversion rates and growth.

Key gaps were identified, including the lack of specific studies and insufficient information about technical parameters and encapsulating agents. The ionic gelation technique was predominant, noted for its effectiveness in protecting bioactive compounds. The most commonly used bioactive substances include carvacrol, thymol, citral, and thyme essential oil and organic acids such as hexanoic, benzoic, butyric, fumaric, and sorbic acids. There is a need for more detailed studies on encapsulation techniques, diversification of animal models, and analysis of long-term effects. Future research may further solidify the sustainable use of these additives, enhancing the animal production potential.
